# Significantly greater triglyceridemia in Black African compared to White European men following high added fructose and glucose feeding: a randomized crossover trial

**DOI:** 10.1186/s12944-016-0315-3

**Published:** 2016-09-02

**Authors:** Louise M. Goff, Martin B. Whyte, Miriam Samuel, Scott V. Harding

**Affiliations:** 1 Division of Diabetes and Nutritional Sciences, King’s College London, Franklin-Wilkins Building, Stamford Street, London, SE1 9NH UK; 2 Department of Diabetes and Metabolic Medicine, University of Surrey, Wolfson Unit for Translational Research, Postgraduate Medical School, Daphne Jackson Road, Guildford, GU2 7WG UK; 3 Division of Diabetes and Nutritional Sciences, King’s College London, Henriette Raphael Building, Room 2.29, Guy’s Campus, London, SE1 1UL UK

**Keywords:** Ethnicity, Fructose, Insulin, Triglycerides, Postprandial

## Abstract

**Background:**

Black African (BA) populations are losing the cardio-protective lipid profile they historically exhibited, which may be linked with increasing fructose intakes. The metabolic effects of high fructose diets and how they relate to blood lipids are documented for Caucasians, but have not been described in BA individuals.

**Objective:**

The principle objective of this pilot study was to assess the independent impacts of high glucose and fructose feeding in men of BA ancestry compared to men of White European (WE) ancestry on circulating triglyceride (TG) concentrations.

**Methods:**

Healthy males, aged 25–60 years, of BA (*n* = 9) and WE (*n* = 11) ethnicity were randomly assigned to 2 feeding days in a crossover design, providing mixed nutrient meals with 20 % total daily caloric requirements from either added glucose or fructose. Circulating TG, non-esterified fatty acids (NEFA), glucose, insulin and C-peptide were measured over two 24-h periods.

**Results:**

Fasting TGs were lower in BAs than WEs on the fructose feeding day (*p* < 0.05). There was a trend for fasting TG concentrations 24 h following fructose feeding to increase in both BA (baseline median fasting: 0.80, IQR 0.6–1.1 vs 24-h median post-fructose: 1.09, 0.8–1.4 mmol/L; *p* = 0.06) and WE (baseline median fasting 1.10, IQR 0.9–1.5 vs 24-h median post-fructose: 1.16, IQR 0.96–1.73 mmol/L; *p* = 0.06). Analysis within ethnic group demonstrated that in TG iAUC was significantly higher in BA compared to WE on both glucose (35, IQR 11–56 *vs* −4, IQR −10–1 mmol/L/min; *p* = 0.004) and fructose (48, IQR 15–68 *vs* 13, IQR −7–38 mmol/L/min; *p* = 0.04). Greater suppression of postprandial NEFA was evident in WE than BA after glucose feeding (−73, IQR −81– −52 *vs* −26, IQR −48– −3 nmol/L/min; *p* = 0.001) but there was no ethnic difference following fructose feeding.

**Conclusions:**

Understanding the metabolic effects of dietary acculturation and Westernisation that occurs in Black communities is important for developing prevention strategies for chronic disease development. These data show postprandial hypertriglyceridemia following acute feeding of high added fructose and glucose in BA men, compared to WE men, may contribute to metabolic changes observed during dietary acculturation and Westernisation.

**Trial registration:**

The study was retrospectively registered on clinicaltrials.gov: NCT02533817.

**Electronic supplementary material:**

The online version of this article (doi:10.1186/s12944-016-0315-3) contains supplementary material, which is available to authorized users.

## Background

Ethnic-specific metabolic effects of fructose have not been described despite comprehensive literature describing the deleterious effects of high fructose diets. Populations of African ancestry have historically been characterised by a distinctly cardio-protective fasting lipid profile (low total and LDL-cholesterol, high HDL-cholesterol and low TG) [1, 2] and low rates of myocardial infarction (MI) [3–5]. However recent evidence recognises a changing profile of cardiovascular morbidity in young Blacks, with increasing rates of MI [6, 7], and fasting lipids that are no longer different to Whites [8]. These changes are not explained by clinical or socioeconomic factors suggesting that lifestyle alterations may be playing a crucial role [6, 9]. Dietary acculturation of migrant populations, from a traditional diet to one that includes ‘Westernised’ foods is believed to play a role in the changing health profile and is characterised by high intakes of biscuits/cookies, cakes, sugar-sweetened beverages and confectionary [10] leading to increased fat, saturated fat and sugar intakes [11, 12]. Acculturation and adoption of Western dietary patterns increases with the length of time since migration, and becomes more apparent in subsequent generations [13]. To this effect, within the UK population people of Black Caribbean ancestry show greater dietary acculturation than people of Black African ancestry whose migration is more recent [14]. In line with this Black Caribbeans exhibit fasting lipids that are no different to Whites however Black Africans still exhibit cardioprotective lipids [8], which may be lost as they adopt more Westernised dietary patterns.

The use of high-fructose corn syrup (HFCS) in the food industry and manufacture of sugar-sweetened beverages and confectionary has received much attention in recent years. Due to increased intake of products containing HFCS and sucrose the fructose load of the Western diet has increased significantly in recent decades [15] and it is estimated that two-thirds of dietary fructose is provided by beverages and other manufactured foodstuffs such as confectionary and only a third of fructose intake is from natural sources such as fruit and vegetables [16]. Fructose feeding has been shown to have significant effects on lipid metabolism and regulation. Studies of acute [17–22] and chronic [23] fructose feeding (usually 20–25 % of energy) show that fructose potentiates postprandial lipemia, assessed as significantly increased 24-h post-prandial TG profiles; the mechanisms responsible for these effects are not fully understood but are proposed to include increased hepatic de novo lipogenesis (DNL) and VLDL secretion, and decreased TG clearance [24]. African-Americans consume significantly more sugar and HFCS than Whites and it is estimated that 1 in 6 African-Americans are consuming more than 25 % of their daily calories from added sugars compared to 1 in 11 White-Americans [25].

The metabolic effects of fructose consumption have been relatively well studied in participants of White ethnicity, however to date there are no studies that have examined the impact of high fructose diets in people of African ancestry and explored the role of fructose consumption in the changing cardiometabolic risk profile of Black populations. Using a randomised controlled crossover design we have conducted a pilot investigation to test the hypothesis that acute feeding of high fructose will potentiate significantly greater post-prandial hypertriglyceridemia in men of Black African (BA) origin compared to men of White European (WE) origin.

## Methods

### Design

A randomised crossover trial was conducted to compare the effects of 24 h high added fructose and high added glucose feeding (20 % of daily calories) in men of BA compared with men of WE origin. Fasting and post-prandial circulating TG, non-esterified fatty acids (NEFA), glucose, insulin and C-peptide were measured in 9 BA and 11 WE participants over a 24-h period on two separate days, during which they consumed three isocaloric, mixed nutrient meals accompanied by beverages sweetened with either 100 % fructose or 100 % glucose (Additional file 1: Table S1), in a randomised crossover fashion.

### Participants

Healthy men (age range: 25–60 y, BMI range: 18–35 kg/m^2^) of WE (*n* = 11) and BA (*n* = 9) origin were recruited from the general population via research recruitment emails and newspaper advertisements. Potential participants attended the research clinic for screening of eligibility and were excluded if found to have fasting glucose in the diabetic range (fasting glucose >7 mmol/L), or found to have hypertension or hyperlipidaemia deemed to require immediate medical intervention (defined as: total cholesterol >6.0 mmol/l; LDL-cholesterol >4.5 mmol/L; fasting TG >3 mmol/L).

Ethnicity was both self-defined by the participants, and confirmed by records of parental and grandparental origin and birthplace; WE participants had at least 3 grandparents originating from countries of Europe and BA participants had at least 3 grandparents originating from countries of West Africa (Economic Community of West African States (ECOWAS) and Central African countries (e.g. Cameroon, Uganda, etc.).

The study was approved and conducted according to the standards of the King’s College London Research Ethics Committee (BDM-12-13-7), and written informed consent was provided by all participants. A small remuneration was provided to acknowledge the participants’ time and commitment. Data collection was conducted January 2013 – July 2014. The study is registered on clinicaltrials.gov: NCT02533817.

### Experimental protocol

Each participant attended the research unit for two controlled experimental feeding days, with a washout period of 21–28 days between visits. The order of the feeding days was randomly assigned using sealed envelopes. Prior to each feeding day participants were instructed to avoid strenuous exercise and alcohol consumption in the preceding 48 h and fast and abstain from smoking from 10 pm the evening before their visit. Participants were provided with a standardised meal for their evening meal prior to their visit, which provided approximately 700kcals, with a macronutrient profile of approximately 55 % carbohydrate, 30 % fat, 15 % protein. Participants attended the research centre at 8 am having consumed nothing other than water since 10 pm the evening prior. During each experimental feeding day the participants were present at the research centre from 8 am until 4 pm and consumed a standardised 24-h menu that was identical for both visits. Participants were instructed to consume only the foods and drinks provided by the research team in the 24 h of study. Participants returned to the research unit in a fasting state at 24 h for their final blood sample after which they were provided with a buffet breakfast of free choice. Participants were required to maintain sedentary behaviour whilst in the research unit and were asked to refrain from physical activity during the 24-h protocol.

Blood sampling was performed using an 18G cannula inserted into the ante-cubital fossa vein of the non-dominant arm which was kept patent with saline flushes. A series of three fasting baseline blood samples were drawn at −30, −15 and 0 min. During the baseline period the following anthropometric measurements were performed: body weight (kg) was measured in light clothing without shoes using digital scales (Tanita BC418MA, Toyko, Japan), height was measured to the nearest 0.5 cm (without shoes) using a wall-mounted stadiometer, waist circumference was measured using a flexible tape at the mid-point between the lowest rib and the iliac crest (mean of three measurements) and body composition including body fat percentage, was assessed by bioelectrical impedance (Tanita BC418MA, Toyko, Japan). Seated blood pressure was measured after a 10 min rest (mean of three measurements). At time 0 min participants were provided with a study breakfast (Additional file 1: Table S1) which they were required to consume within a 15 min period and subsequent blood samples were drawn at 15, 30, 45, 60, 90, 120, 180, 240, 300, 360 and 420 min. The lunch meal was consumed immediately following the 240 min blood sample and final fructose/glucose drink was consumed immediately following the 420 min blood sample. A final blood sample was drawn at 24-h under fasting conditions. Each blood sample involved the removal of 2 mL of ‘dead space’ to clear the catheter tubing followed by a 10 mL sample into blood collection tubes containing no anticoagulant or EDTA, lithium heparin, fluoride oxalate. Samples were centrifuged (following 20 min clotting time for serum), divided into aliquots and stored at −80 °C until assayed.

### Meals

The menus were developed by a research dietitian to provide an energy profile of approximately 50 % carbohydrate, 35 % fat and 15 % protein, consisting of whole foods and commercially available dishes. The nutrient composition of the menus was analysed using Food Processor software (ESHA Research, Salem, OR). The total calorie content of the menu for each individual participant was based on an estimation of the their energy requirements, which was calculated using standard equations for basal metabolic rate [26] and multiplied by a moderately sedentary physical activity ratio (1.4); these requirements were rounded up to the nearest standardised block (2200, 2500, 2900, 3200 kcals/day). The menu was divided into 3 meals of approximately equal energy content, breakfast provided 28 % of daily calories, lunch 36 % and the evening meal 35 %. With each meal a drink containing either fructose or glucose powder was consumed which provided 25 % of daily calories at breakfast and 18 % of daily calories at each of the other meals, totalling 20 % of calories in the 24-h period provided by added fructose or glucose; lemon flavouring was used to disguise the sweetness of the drinks. All other menu components were identical on both feeding days and participants were provided with water to drink but no other beverages (Additional file 1: Table S1). The total quantity of added glucose or fructose ranged from 110 g for the 2200 kcals menu to 161 g for the 3200 kcals menu. Participants were instructed to consume the fructose/glucose drink at the beginning of each meal before consuming the food dishes and instructed to consume all the foods provided. The breakfast meal was served at approximately 9.30 am, lunch was served 4 h later and the evening meal was packaged for participants to consume in their own home, although all the fructose/glucose drinks were consumed before the participants departed the research unit, in the presence of the research team.

### Laboratory analyses

Serum triglycerides, NEFA and glucose, as well as baseline serum total and HDL cholesterol, were measured by colour photometric assays on an automated clinical chemistry analyser (ILab 650, Instrument Laboratories); serum LDL-cholesterol was calculated by Friedwald equation. Serum insulin and C-peptide were determined by automated chemiluminescent immunoassay (Immulite 2000 XPi).

### Data analysis

Postprandial TG, NEFA, glucose, insulin and C-peptide were expressed as the area under the curve (AUC), analysed using trapedzoidal rule in Sigmaplot 12.3 (Systat Software, Inc. San Jose, CA, USA). To more accurately describe the response to feeding, the incremental AUCs (iAUC) were calculated by correction for the baseline (fasting) timepoint [27, 28]. Missing values for a given time-point (<1.5 % of all the glucose and TG values) were imputed by interpolation. The iAUC was calculated for 0–180 mins, to measure the first meal effect, and also 0–420 mins to measure the cumulative effect of first and second meals, and importantly to recognise the different post-prandial patterns of blood glucose and triglycerides.

Insulin resistance was estimated by homeostasis model assessment (HOMA-IR), calculated as [mean fasting insulin (mU/mL) × mean fasting glucose (mmol/L)]/22.5 [29].

Data that failed the Shapiro-Wilk normality test (plasma TG concentration) were log transformed prior to analysis. A 2-way ANOVA analysis was used to assess differences by ethnicity and treatment for biochemical time-points and iAUC comparisons. Because of the different postprandial metabolism (insulin-dependent versus non-insulin-dependent) of the 2 treatments and that glucose is an active comparator, not true control, we conducted additional statistical analysis. Comparisons between treatments within each ethnic group individually were made by paired *t*-test and comparisons between ethnic groups for each treatment (individual time points and iAUC for each outcome) were made by two-tailed independent samples *t*-test with Sidek’s correction for multiple testing. Otherwise described, all data were analysed using SPSS IBM version 21 (SPSS Inc., Chicago, IL, USA).

## Results

### Baseline characteristics

The characteristics of the participants are shown in Table 1. Within the BA group 6 (66 %) participants were born in the UK and had resided in the UK all of their lives and 3 (33 %) participants were migrants and had resided in the UK for 10 years or longer. There were no significant differences in anthropometric and baseline characteristics between ethnic groups (Table 1), although there was a trend towards higher diastolic blood pressure in BAs. Grade 1 hypertension (140–159/90–99 mmHg) was present in two WE and one BA, isolated systolic hypertension (grade 1, 140–159/<90 mmHg) was evident in one WE participant, grade 2 hypertension (160–179/100–109 mmHg) in one BA and grade 3 hypertension (≥180/≥110 mmHg) in one BA. Mean BMI for both groups was within the overweight range (BMI > 25) [30].

**Table 1:** Baseline anthropometric and serum biochemical characteristics of Black African and White European participants

	BA	WE
N	9	11
Age, years	38.3 ± 2.0	34.7 ± 2.2
Body weight, kg	87.5 ± 4.9	80.9 ± 4.0
BMI, kg/m^2^	27.5 ± 1.4	25.0 ± 1.1
WC, cm	88.4 ± 3.1	90.9 ± 2.3
Body fat, %	19.5 ± 1.9	19.7 ± 2.1
Systolic blood pressure, mmHg	139 ± 8	128 ± 6
Diastolic blood pressure, mmHg	87 ± 4*	76 ± 4*
Total cholesterol^a^, mmol/L	4.8 ± 0.3	4.6 ± 0.3
HDL-cholesterol^a^, mmol/L	1.3 ± 0.1	1.4 ± 0.1
LDL-cholesterol^a^, mmol/L	2.9 ± 0.2	2.7 ± 0.2
Triglycerides^a^, mmol/L	1.09 ± 0.15	1.45 ± 0.18
HOMA-IR^b^	0.93 ± 0.31	0.82 ± 0.22

*Abbreviations*: *BA* Black African, *BMI* body mass index, *HDL* high density lipoprotein cholesterol, *HOMA-IR* homeostatic model assessment of insulin resistance, *LDL* low density lipoprotein cholesterol, *WC* waist circumference, *WE* White European

Data are mean ± SEM. *n* = 9 (BA) and *n* = 11 (WE). *P < 0.1; differences between ethnic group tested by independent samples *t*-test

^a^Based on non-fasted serum sample

^b^Based on fasted serum samples

### Fasting triglycerides

Fasting TGs were lower in BAs than WEs on the fructose feeding day (Table 2) and neared statistical significance on the glucose feeding day. There were no other fasting differences between ethnicities.

**Table 2:** Fasting and postprandial glucose, NEFA, triglyceride, C-peptide and insulin response to fructose-rich or glucose-rich meals in Black African and White European participants

	BA	WE
Glucose-rich meals		
Fasting triglyceride, mmol/L	0.84 (0.61–1.20)^a^	1.18 (0.78–1.41)^a^
Fasting triglyceride 24 h, mmol/L	0.97 (0.75–1.39)	1.41 (0.87–1.51)
Triglyceride iAUC_0–180,_ mmol/L/min	35 (11–56)^a^	−4 (−10–1)^a,b^
Triglyceride iAUC_0–420,_ mmol/L/min	268 (77–334)^a^	39 (0–111)^a, b^
Fasting glucose, mmol/L	4.9 ± 0.3	5.0 ± 0.2
Glucose iAUC_0–180,_ mmol/L/min	65 (−45–139)	27 (−18–83)
Glucose iAUC_0–420,_ mmol/L/min	40 (−281–251)	96 (−68–143)
Fasting NEFA, μmol/L	0.7 ± 0.1	0.8 ± 0.09
NEFA iAUC_0–180,_ μmol/L/min	−26 (−48– −3)^a^	−72.60 (−81– −52)^a,b^
NEFA iAUC_0–420,_ μmol/L/min	−51 (−93–96)^a^	−148.39 (−164– −94)^a^
Fasting insulin, mU/L	4.4 ± 1.3	3.5 ± 0.8
Insulin iAUC_0–180,_ mU/L/min	3577 (1828–5035)	3596 (1939–4828)
Fasting C-peptide, pmol/L	424 ± 80	409 ± 55
C-peptide iAUC_0–180,_ pmol/L/min	188,000 (112,000–259,000)	208,000 (117,000–276,000)^b^
Fructose-rich meals		
Fasting triglyceride, mmol/L	0.80 (0.6–1.1)^a^	1.10 (0.9–1.5)^a^
Fasting triglycerides 24 h, mmol/L	1.09 (0.80–1.54)	1.16 (0.96–1.73)
Triglyceride iAUC_0–180,_ mmol/L/min	48 (15–68)^a^	13 (−7–38)^a,b^
Triglyceride iAUC_0–420,_ mmol/L/min	217 (139–279)^a^	162 (37–219)^a,b^
Fasting glucose, mmol/L	4.8 ± 0.2	5.1 ± 0.1
Glucose iAUC_0–180,_ mmol/L/min	−2 (−109–50)	−11 (−147–69)
Glucose iAUC_0–420,_ mmol/L/min	−149 (−318–61)	−60 (−204–138)
Fasting NEFA, μmol/L	0.8 ± 0.1	0.7 ± 0.1
NEFA iAUC_0–180,_ μmol/L/min	−33 (−51–12)	−40 (−64– −30)^b^
NEFA iAUC_0–420,_ μmol/L/min	^−^51 (−96–46)	−77 (−124–−10)
Fasting insulin, mU/L	3.3 ± 1.3	3.5 ± 0.5
Insulin iAUC_0–180,_ mU/L/min	1710 (831–3975)	2318 (863–3276)
Fasting C-peptide, pmol/L	329 ± 60	399 ± 49
C-peptide iAUC_0–180,_ pmol/L/min	101,940 (68,708–148,140)	110,340 (80,753–175,901)^b^

*Abbreviations*: *BA* Black African, *iAUC* incremental area under the curve, *NEFA* non-esterified fatty acids, *WE* White European

Data mean ± SEM or median (IQR), *n* = 9 (BA) and *n* = 11 (WE)

^a^Denotes within treatment difference between ethnic group tested by independent samples *t*-test, *P* < 0.05

^b^Denotes within ethnic group difference between treatment tested by paired *t*-test, *P* < 0.05

### Postprandial Triglycerides and NEFA

**Fig. 1:**
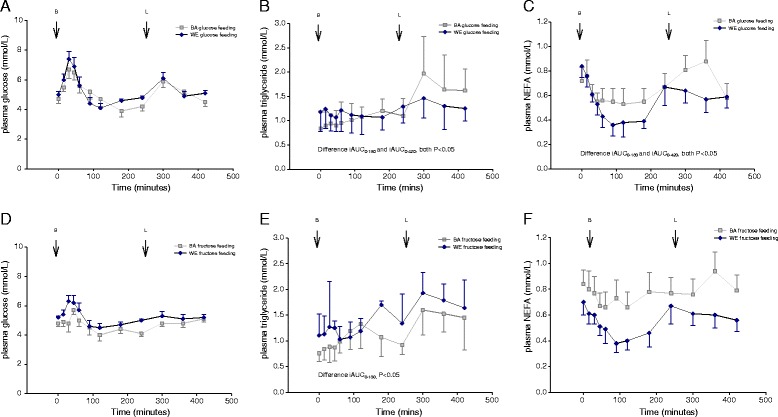
Serum glucose (**a**), TG (**b**) and NEFA (**c**) response to glucose-rich meals in BA (light square) and WE (dark diamonds) participants, and serum glucose (**d**), triglyceride (**e**) and NEFA (**f**) response to fructose-rich meals in BA (light square) and WE (dark diamonds) participants. Breakfast meal consumed at 0 min and lunch meal consumed following the 240 min sample. Point estimates are mean ± SEM for glucose and NEFA, and median (IQR) for triglyceride. Light squares: BA; dark diamonds; WE. BA, Black African; NEFA, non-esterified fatty acids; TG, triglyceride; WE, White European

The 420-min time-course of glucose, TG and NEFA concentrations, after two consecutive glucose-rich or fructose-rich meals, are shown in Fig. 1. The TG iAUC was higher in BAs than WEs at both iAUC_0–180_ and iAUC_0–420_ on the glucose-rich feeding day (Table 2; Fig. 1). Twenty-four hours after glucose feeding the median fasting TG were significantly increased in BA (0.84, IQR 0.61–1.20 *vs* 0.97, IQR 0.75–1.39 mmol/L; *p* < 0.02) but not WE (1.18, IQR 0.78–1.41 *vs* 1.41, IQR 0.87–1.51 mmol/L; *p* = 0.14). On the fructose-rich feeding day TG iAUC_0–180_ was greater in BAs but iAUC_0–420_ was not different (Table 2). Twenty-four hours after fructose feeding, the median fasting TG were no longer different between BAs (1.09, IQR 0.8–1.4 mmol/L) and WEs (1.16, IQR 0.96–1.73 mmol/L; *p* = 0.4). There was a trend for fasting TG concentrations 24 h following fructose feeding to increase in both BA (baseline median fasting: 0.80, IQR 0.6–1.1 *vs* 24-h median post-fructose: 1.09, 0.8–1.4 mmol/L; *p* = 0.06) and WE (baseline median fasting 1.10, IQR 0.9–1.5 *vs* 24-h median post-fructose: 1.16, IQR 0.96–1.73 mmol/L; *p* = 0.06). Analysis within ethnic group demonstrated that in BAs there were no differences in TG iAUC between glucose- or fructose-rich feeding regimen, however in WEs, there were trends towards greater TG iAUC at iAUC_0–180_ (*p* = 0.053) and at iAUC_0–420_ (*p* = 0.076) with fructose-rich feeding than with glucose-rich feeding. In both ethnic groups the NEFA concentration following feeding was suppressed to levels below the fasting value, leading to negative AUCs at 180 and 420 min (Table 2; Fig. 1). Greater suppression of postprandial NEFA was evident in WEs after glucose-rich feeding than in BAs at iAUC_0–180_ and iAUC_0–420_. No ethnic difference in NEFA suppression was seen after fructose-rich feeding.

### Postprandial glucose

**Fig. 2:**
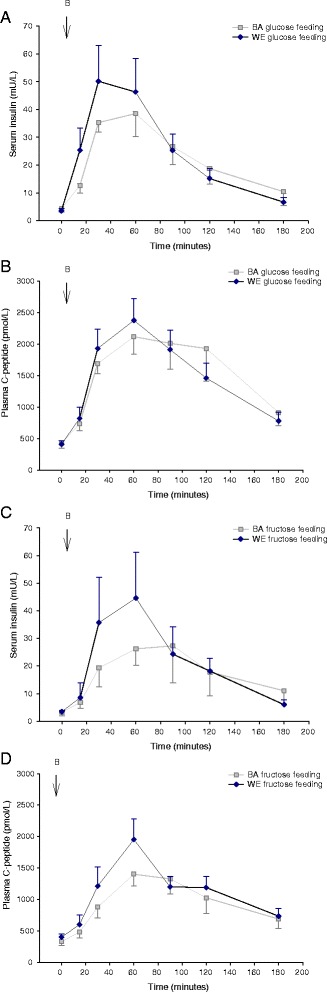
Serum insulin (**a**) and C-peptide (**b**) response to glucose-rich meals in BA (light square) and WE (dark diamonds) participants, and serum insulin (**c**) and C-peptide (**d**) response to fructose-rich meals in BA (light square) and WE (dark diamonds) participants. Breakfast meal consumed at 0 min. Point estimates are mean ± SEM. Light square: BA; dark diamonds: WE. BA, Black African; WE, White European

There were no differences between ethnicities in peak glucose concentration or glucose iAUC (Table 2; Fig. 1). In WEs, peak glucose was at 45-min with glucose-rich feeding and 30 min with fructose-rich feeding and there were no differences in plasma glucose concentration at any time point. In BAs, peak glucose was after 30-min with glucose-rich feeding and at 45-min with fructose-rich feeding. In BAs, plasma glucose was greater at 120-min (4.72 ± 0.48 vs 4.04 ± 0.41 mmol/L; *P* = 0.003) and at 300-min (5.91 ± 0.38 *vs* 4.83 ± 0.23 mmol/L; *P* = 0.004) with glucose-rich feeding compared to fructose-rich feeding. C-peptide and insulin concentrations were not different, between ethnicities, on either feeding day (Table 2; Fig. 2); specifically, the peak response, incremental response and iAUC were similar. As expected, in both ethnic groups, glucose-rich feeding led to higher insulin and C-peptide responses than fructose-rich feeding (Fig. 2). Peak C-peptide response occurred after 60 min in both ethnic groups and with both types of feeding. Peak insulin occurred after 30-min in both ethnicities with a glucose-rich meal but after 60-min with a fructose-rich meal. There was no significant interaction between ethnicity and feeding regimen, tested by two-way ANOVA, on glucose or lipid responses to glucose-rich or fructose-rich feeding.

## Discussion

To our knowledge this pilot investigation is the first to examine ethnic specific effects of fructose feeding and demonstrates markedly greater TG excursions in BA participants compared to WEs in response to high fructose and high glucose intakes, despite significantly lower fasting TGs.

Low fasting TG concentrations have been extensively reported in populations of African ancestry [1, 31, 32] and are proposed to contribute to the protection from coronary artery disease (CAD) that they exhibit [4, 5]. However recent data have demonstrated that this cardio-protection has been lost amongst younger populations [6, 7] and Blacks no longer exhibit lower TGs than Whites [8]. In our study, BA men were found to have lower fasting TGs at baseline but not different to the WEs 24 h post fructose treatment (Table 2). This suggests that consumption of high fructose loads may have detrimental effects on fasting lipids in people of African ancestry. Traditionally the African and Caribbean diet is not high in added sugar [33] however recent USA data suggests that consumption of added sugar is higher amongst African-Americans than Whites [25], and in our own UK data we have demonstrated that in Black Caribbean adults sugar-sweetened beverages and confectionary are the 5th and 8th most important contributors to energy intake, respectively [14]. Interestingly ‘Westernisation’ of the diet is much less evident in those whose migration is more recent [14] and indeed these less acculturated communities still exhibit lower fasting TGs [8] and may still be protected from CAD. The majority of the BA participants in the present study were born in the UK, and those who weren’t had resided in the UK for a significant length of time, therefore their diet is likely to have undergone significant acculturation. However, the lower fasting triglycerides that were evident suggest there is still some level of cardioprotection amongst this group.

Post-prandial lipid handling has not been previously investigated in this ethnic group despite the growing body of evidence to show their importance as indicators of a proatherogenic state [34, 35]. Our data suggest that in response to both glucose and fructose BAs exhibited a marked hypertriglyceridemia, which may help to explain some of the detrimental health outcomes associated with consumption of high sugar diets. We are not able to determine the exact mechanisms driving this hypertriglyceridemic response, for example the relative contributions of increased hepatic secretion of TG-rich very low density lipoprotein (VLDL) and decreased TG clearance (predominantly into adipose tissue). Recent tracer studies have shown increased chylomicron production rates in patients with the metabolic syndrome [36] but to our knowledge, no comparative data exist of chylomicron production rates between ethnicities. It is noteworthy that following glucose feeding suppression of NEFA was significantly less in the BA than the WE participants potentially providing greater substrate availability for VLDL-TG secretion. The reduced NEFA suppression by the BA participants, despite similar insulin secretion and circulating insulin concentrations, allows us to speculate that there is a greater adipocyte insulin resistance in the BAs, despite no differences in HOMA index of whole body insulin resistance between ethnicities. If greater adipocyte insulin resistance is indeed the case it may have led to reduced TG clearance, contributing to the hypertriglyceridaemic response that we observed.

The strengths and limitations of our study warrant consideration. This investigation was a pilot study involving a small number of participants however we used tightly controlled conditions to study the ethnic specific metabolic effects of acute high fructose and glucose feeding. Our participants were closely matched with no differences in age, BMI or waist circumference, which are important determinants of lipid metabolism and cardiovascular risk and therefore we are confident that our differences are mainly ethnicity based. We do however acknowledge the broad range in age and BMI of our participants, which may have contributed to heterogeneity in our data. Our BA participants exhibited higher blood pressure and lower fasting TGs, which was to be expected given the extensive literature that reports this cardiometabolic profile amongst people of African ancestry [37, 38]. Our feeding protocol was informed by, and comparable with the literature [21, 22]; it provided 20 % of daily energy as added glucose or fructose in the form of a drink, which was ingested alongside a menu of usual dietary items that provided an overall nutrient composition (approximately 50 % carbohydrate, 35 % fat and 15 % protein) similar to the UK diet and was not expected to invoke significant post-prandial lipemia. Our participants were provided with a standardised meal for the evening meal preceding the feeding days in an attempt to standardise the ‘second meal’ metabolic effect. In agreement with the literature [22, 39] we demonstrated a significantly lower glucose, insulin and C-peptide response to fructose feeding compared to glucose, which gives us confidence in the design of our protocol; furthermore the participants consumed the menus in the presence of the investigators and therefore we are confident that we achieved a high level of compliance. Our sample size was small and our protocol studied only short term effects of high sugar consumption, however we believe we have generated persuasive initial data to demonstrate ethnicity specific metabolic effects that warrant further investigation in a large scale, longer term trial. This study did not involve use of isotope tracer techniques which would have enabled us to investigate in detail the mechanisms by which dietary sugars promote hypertriglyceridemia and future investigations should include these methods. Additionally stable isotope studies with adipose and muscle tissue biopsies and more in depth analysis of body composition, particularly visceral fat deposits, could be used to investigate further our hypothesis that the failure to suppress NEFAs amongst the BA participants may be as a result of intensified adipocyte insulin resistance.

## Conclusions

In conclusion these pilot data illustrate the short term metabolic effects of acute high added fructose and high added glucose feeding in healthy men of BA ethnicity. In particular, this study reports significant postprandial hypertriglyceridemia in men of BA ethnicity compared to men of WE ethnicity. These subtle but distinct ethnic differences in the postprandial handing of high added sugar is an important consideration for future study of ethnic specific risk in disease cardiometabolic disease development. Finally, while preliminary, these data describe a metabolic response to high sugar feeding in the postprandial period that may influence the process of dietary acculturation and Westernisation, which occurs in Black communities. Therefore, the postprandial response to high fructose and glucose feeding is important to consider in the study of cardiometabolic risk development in different ethnicities.

## Additional file

Additional file 1: Nutritional composition of the experimental feeding day standardised menu (2500 kcal/day), providing 20 % energy as added glucose or fructose. (DOCX 12 kb)

## Abbreviations

BA: Black African
BMI: Body mass index
CAD: Coronary artery disease
MI: Myocardial infarction
NEFA: non-esterified fatty acids
TG: triglycerides
WC: waist circumference
WE: White Europeans
