# Supplementary material for: Significantly greater triglyceridemia in Black African compared to White European men following high added fructose and glucose feeding: a randomized crossover trial
Source: Lipids Health Dis. 2016 Sep 2;15(1):145. doi: 10.1186/s12944-016-0315-3 (PMC5009494; doi:10.1186/s12944-016-0315-3)
Supplement: Additional file 1: — Nutritional composition of the experimental feeding day standardised menu (2500 kcal/day), providing 20 % energy as added glucose or fructose. (DOCX 12 kb) [file 12944_2016_315_MOESM1_ESM.docx]

**Table S1. Nutritional composition of the experimental feeding day standardised menu (2500 kcal/day), providing 20% energy as added glucose or fructose**

| Menu | Nutrient composition |  |
| --- | --- | --- |
|  |  |  |
| Breakfast |  |  |
| 42g Fructose or glucose drink (350mL) | Energy, kcal | 710 |
| 100g Scrambled eggs | CHO, g (% energy) | 101g (53) |
| 100g White bread, toasted | Fat, g (% energy) | 26g (33) |
| 12g Margarine (70% fat) | Protein, g (% energy) | 21g (12) |
| 50mL Semi-skimmed milk |  |  |
| 250 mL Decaffeinated tea *or* coffee |  |  |
| Lunch |  |  |
| 42g Fructose or glucose drink (350mL) | Energy, kcal | 893 |
| 280g Macaroni cheese | CHO, g (% energy) | 119g (50) |
| 20g Green salad | Fat, g (% energy) | 34g (34) |
| 30mL Low fat salad dressing | Protein, g (% energy) | 29g (13) |
| 110g Yogurt |  |  |
| 50mL Semi-skimmed milk |  |  |
| 250 mL Decaffeinated tea *or* coffee |  |  |
| Evening meal |  |  |
| 42g Fructose or glucose drink (350mL) | Energy, kcal | 888 |
| 400g Chicken & bacon pasta bake | CHO, g (% energy) | 111g (47) |
| 20g Green salad | Fat, g (% energy) | 35g (35) |
| 90g Rice pudding | Protein, g (% energy) | 34g (15) |
